# COVID-19 Vaccination Willingness Among People Living With HIV in Wuhan, China

**DOI:** 10.3389/fpubh.2022.883453

**Published:** 2022-05-09

**Authors:** Songjie Wu, Fangzhao Ming, Zhongyuan Xing, Zhiyue Zhang, Shanhui Zhu, Wei Guo, Shi Zou, Jinli Liu, Yang Liu, Ke Liang

**Affiliations:** ^1^Department of Nosocomial Infection Management, Zhongnan Hospital of Wuhan University, Wuhan, China; ^2^Hubei Engineering Center for Infectious Disease Prevention, Control and Treatment, Wuhan, China; ^3^Wuchang District Center for Disease Control and Prevention, Wuhan, China; ^4^School of Basic Medical Sciences, Wuhan University, Wuhan, China; ^5^Medical Department, Zhongnan Hospital of Wuhan University, Wuhan, China; ^6^Department of Pathology, Zhongnan Hospital of Wuhan University, Wuhan, China; ^7^Department of Pathology, School of Basic Medical Sciences, Wuhan University, Wuhan, China; ^8^Department of Infectious Diseases, Zhongnan Hospital of Wuhan University, Wuhan University, Wuhan, China; ^9^Zhongnan Hospital of Wuhan University, Wuhan University, Wuhan, China; ^10^School of Economics and Management, Wuhan University, Wuhan, China; ^11^Wuhan Research Center for Infectious Diseases and Cancer, Chinese Academy of Medical Sciences, Wuhan, China

**Keywords:** COVID-19 vaccination, willingness, PLWH, general population, Wuhan

## Abstract

Vaccination is essential to controlling the pandemic of coronavirus disease 2019 (COVID-19). People living with HIV (PLWH) were considered more vulnerable to the severe acute respiratory syndrome coronavirus 2 (SARS-CoV-2) infection compared with the general population. Therefore, it is urgent to protect PLWH from SARS-CoV-2 infection. For PLWH, vaccine hesitancy could be more common and may compromise vaccine coverage. Our study aimed to investigate the willingness to receive the COVID-19 vaccination among PLWH and associated factors. A cross-sectional online survey was performed among PLWH and the general population from 4 April to 18 April 2021 in Wuhan, China. The multivariable logistic regression was used to analyze associated factors for COVID-19 vaccination willingness among PLWH. A total of 556 PLWH and 570 individuals from the general population were enrolled. The COVID-19 vaccine willingness among PLWH was 60.8%, which was relatively lower than that in the general population (80.9%) (*P* < 0.001). The results of multivariable analysis indicated that PLWH with comorbidities (OR = 2.07, 95% CI: 1.25–3.45), those who had idea about PLWH would be more serious if they were infected with SARS-CoV-2 (OR = 1.67, 95% CI: 1.11–2.51) and those who thought their antiretroviral therapy (ART) would be affected by COVID-19 epidemic (OR = 2.04, 95% CI: 1.22–3.42) had higher willingness to receive COVID-19 vaccination. PLWH who had a monthly income over 5,000 RMB (OR = 0.64, 95% CI: 0.45–0.92) and had a sex orientation as non-homosexual (OR = 0.67, 95% CI: 0.47–0.96) were associated with lower willingness for COVID-19 vaccination. Our findings showed that the PLWH had a lower willingness for COVID-19 vaccination compared with the general population in Wuhan. Targeted interventions such as health education should be conducted to increase the willingness for COVID-19 vaccination among PLWH, thus enhancing COVID-19 vaccine uptake among PLWH.

## Introduction

The coronavirus disease 2019 (COVID-19), which spread rapidly worldwide to become a global pandemic resulting to more than 424 million infections and 5.8 million deaths up to 22nd Feb 2022 (1). COVID-19 bring more challenges for people living with HIV (PLWH), studies have shown that PLWH were more likely to be infected with severe acute respiratory syndrome coronavirus 2 (SARS-CoV-2) (2) and had poorer outcomes (3, 4) compared with HIV-uninfected individuals. In addition to the physical impact, the COVID-19 pandemic also affected the emotional and social wellbeing of PLWH, which brought more obstacles for the health care and the access to antiretroviral therapy (ART) (5). So, extensive public health measures should be implemented to reduce further transmission of SARS-CoV-2, especially for vulnerable populations including PLWH.

Vaccination would be one of the most important measures for controlling the epidemic of COVID-19. With the global popularity of the COVID-19 vaccination, evidence has shown that the COVID-19 vaccines could reduce the morbidity and mortality of the disease effectively (6). Simulation experiments showed that 75% coverage of vaccination could control the COVID-19 pandemic without any other control measures when vaccine efficacy was 80% (7). A number of studies observed a comparable immune response and safety between PLWH and HIV-negative individuals in response to messenger RNA (mRNA) and adenovirus vector COVID-19 vaccines (8, 9). Similar results were also found in inactivated COVID-19 vaccine (10). Our former study also showed no serious adverse events for COVID-19 vaccination were observed among PLWH in China (11). Above evidence had proved that PLWH could benefit from COVID-19 vaccination. However, vaccine hesitancy which could be common, especially in some particular groups including PLWH, which may compromise the coverage of vaccination (12). Therefore, it is crucial to understand the willingness to receive COVID-19 vaccination and related factors among PLWH. Two published studies investigated COVID-19 vaccine willingness among PLWH in the United States (13) and France (14) showed that around 70% participants were willing to receive COVID-19 vaccination. A survey in eight cities (not including Wuhan) of China reported 57.2% PLWH have willingness to COVID-19 vaccination (15).

In China, the COVID-19 pandemic had been well-controlled (16, 17), several factors (18) including high population density and general susceptibility makes the vast majority of Wuhan's residents with no immunity against SARS-CoV-2 and at continued risk of infection. For preventing the potential re-emergence of COVID-19, the Chinese government is also trying to promote the coverage of COVID-19 vaccination, especially for particular groups including PLWH. In the central Chinese city of Wuhan, where the first outbreak of COVID-19 occurred, the COVID-19 vaccination willingness and associated factors among PLWH is unclear. This study aimed to investigate the willingness to receive the COVID-19 vaccine and associated factors among PLWH.

## Materials and Methods

### Study Design and Participants

This cross-sectional study was performed from 4 April to 18 April 2021 among PLWH and individuals from the general population by distributing an online structural questionnaire *via* an investigation platform named Wenjuanxing (www.wjx.cn). PLWH managed by the Wuchang district center for disease control and prevention (CDC) were eligible for recruitment. Individuals from the general population were recruited from the physical examination center in Zhongnan Hospital of Wuhan University. HIV infection was excluded by HIV antibody screening. A link to access an online self-administered questionnaire was sent to the consented participants. All the participants were ≥18 years old.

### Questionnaire and Measures

The questionnaire consists of four sections: (1) sociodemographic information including age, gender, education, occupation, marital status, monthly income; (2) the perception of susceptibility and severity; (3) health status characteristics including comorbidities, history of COVID-19 for themselves or their family members/friends, and other vaccine experiences in the last 3 years; (4) willingness to receive COVID-19 vaccination and reasons for willingness or unwillingness to receive the vaccination.

For PLWH, they were also asked if their ART was affected by the COVID-19 epidemic. And other information including gender identification, sexual orientation, sexual orientation disclosure, ART, HIV viral load, the last CD4 count were collected *via* the Acquired Immune Deficiency Syndrome (AIDS) Comprehensive Prevention and Control Data Information Management System of the Chinese Center for Disease Control and Prevention (CDC).

### Statistical Analysis

Normal distribution of continuous variables was expressed as mean ± standard deviation and tested by the student's *t*-test or variance analysis, while the continuous variables with abnormal distribution were presented as median (interquartile range) and tested by the Wilcoxon rank-sum test. Categorical variables were expressed as *n* (%) and compared by the chi-square test or Wilcoxon rank-sum test. A binary logistic regression model was adopted to test the association of sociodemographic characteristics, health status characteristics, the perception of COVID-19 susceptibility and severity and other variables with the willingness to receive COVID-19 vaccination (dichotomous variable, we defined “Yes = 1” and “No = 0”) among PLWH. The associations between the potential associated factors and willingness to receive the COVID-19 vaccine were presented as odds ratios (ORs) and 95% confidence intervals (CIs). A two-tailed *p*-value < 0.05 was considered statistically significant. SPSS version 26 (IBM Corp., Armonk, NY) was used in the data analysis.

## Results

### Characteristics of Study Participants

In total, 556 PLWH and 570 individuals from the general population were enrolled in this study. Of PLWH, the age (mean ± SD) was 37.59 ±11.45years, 92.6% (515/556) were male, 71.0% (395/556) were unmarried, 13.5% (75/556) had comorbidities (hypertension, diabetes, etc.). About one-half (52.2%, 290/556) of the PLWH self-reported a sexual orientation as homosexual, 39.9% (222/556) had the last CD4+ T lymphocyte count (CD4 count) higher than 500/μL, 96.0% were receiving ART, 73.7% were virally suppressed. Besides, more than four-fifths of the participants ever disclosed (35.6%, 198/556) their sexual orientation to others. The PLWH were younger than the general population group (*P* = 0.001), they were also predominantly male (*P* < 0.001) and had more comorbidities than the general population group (*P* = 0.002). The differences between the two groups in the marital status, education level, occupation, and monthly income were also statistically significant (*P* < 0.05) (Table 1).

**Table 1:** Descriptive characteristics of all participants in Wuhan, China, 2021 (*N* = 1,126).

**Characteristics**	**PLWH (*N* = 556)**	**General population (*N* = 570)**	**Statistics**	***P***
Gender				
Male	515 (92.6)	251 (44.0)		
Female	41 (7.4)	319 (56.0)	305.54	<0.001
Age (Mean ± SD)	37.59 ± 11.45	35.29 ± 12.25	3.26	0.001
Age group				
18–59	523 (94.1)	542 (95.1)		
≥60	33 (5.9)	28 (4.9)	0.58	0.448
Marital status				
Married	161 (29.0)	329 (57.7)		
Single or divorced or widowed	395 (71.0)	241 (42.3)	94.73	<0.001
Education level				
High school or lower	315 (56.6)	154 (27.0)		
Higher than high school	241 (43.4)	416 (73.0)	101.72	<0.001
Occupation				
Medical staff	7 (1.3)	164 (28.8)		
Party and government organs and institutions	123 (22.1)	97 (17.0)		
Self-employed, freelance	174 (31.3)	52 (9.1)		
Transportation, express delivery, service industry	12 (2.2)	37 (6.5)		
Teaching staff	30 (5.4)	58 (10.2)		
Student	14 (2.5)	62 (10.9)		
Retired unemployed and underemployed	92 (16.6)	45 (7.9)		
others	104 (18.7)	55 (9.7)	296.15	<0.001
Monthly income (RMB)				
≤ 5,000	289 (52.0)	237 (41.6)		
>5,000	267 (48.0)	333 (58.4)	12.229	<0.001
Comorbidities				
Yes	75 (13.5)	44 (7.7)		
No	481 (86.5)436	526 (92.3)	9.91	0.002
Have taken other vaccines in the last 3 years				
Yes	58 (10.4)	107 (18.8)		
No	498 (89.6)	463 (81.2)	15.66	<0.001
Willing to vaccination				
Yes	338 (60.8)	461 (80.9)		
No	218 (39.2)	109 (19.1)	55.10	<0.001
Sexual orientation				
Homosexual	290 (52.2)	–	–	–
Heterosexual	137 (24.6)	–	–	–
Bisexual	76 (13.7)	–	–	–
Unsure/Other	53 (9.5)	–	–	–
Sexual orientation disclosure				
Yes	198 (35.6)	–	–	–
No	358 (64.4)	–	–	–
ART				
Yes	534 (96.0)	–	–	–
No	22 (4.0)	–	–	–
HIV viral load				
Not detected	410 (73.7)	–	–	–
Detected	146 (26.3)	–	–	–
The last CD4 count				
0–199/ul	33 (5.9)	–	–	–
200~349/ul	93 (16.7)	–	–	–
350~499/ul	168 (30.2)	–	–	–
≥500/ul	222 (39.9)	–	–	–
Not test	40 (7.2)	–	–	–
ART affected by COVID-19				
Yes	401 (72.1)			
No	155 (27.9)			
Ever diagnosed with COVID-19				
Yes	8 (1.4)	–	–	–
No	548 (98.6)	–	–	–
Family members/friends diagnosed with COVID-19				
Yes	39 (7.0)	–	–	–
No	517 (93.0)	–	–	–
HIV positive members are more likely to get infected with SARS-CoV-2				
Yes	207 (37.2)	–	–	–
No	349 (62.8)	–	–	
PLWH would be more serious if they were infected with SARS-CoV-2				
Yes	403 (60.8)	–	–	–
No	153 (27.5)	–	–	–

### Willingness for Receiving the COVID-19 Vaccination Among PLWH

**Figure 1:**
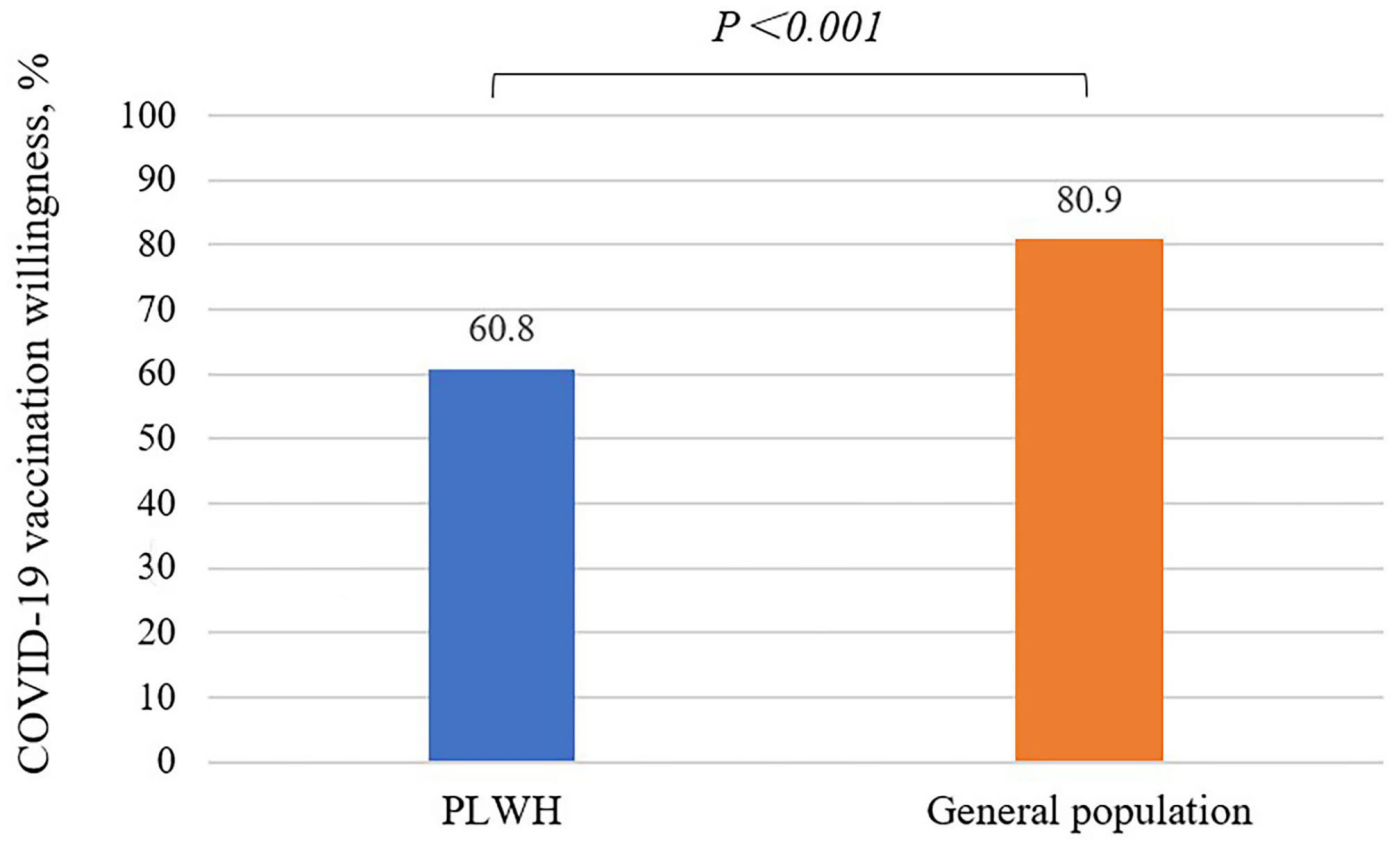
COVID-19 vaccination willingness among PLWH (60.8%) is significantly lower than the general population (80.9%), *P* < 0.001, multivariable logistic regression model was used.

COVID-19 vaccination willingness among PLWH was 65.5%, which was lower than that in the general population group (80.9%). After adjusting the confounding factors (such as age, gender, marital status, educational level, monthly income and comorbidities) with multivariable logistic regression analysis, this difference still existed (*P* < 0.001) (Supplementary Table 1 and Figure 1).

### Factors Associated With Willingness to Receive COVID-19 Vaccination Among PLWH

The results of the logistic regression analyses are shown in Table 2. The PLWH with comorbidities (OR = 2.07, 95% CI: 1.25–3.45), those who thought PLWH would be more serious if they were infected with SARS-CoV-2 (OR = 1.67, 95% CI: 1.11–2.51), and those who thought their ART affected by COVID-19 (OR = 2.041, 95% CI: 1.22–3.42) had higher willingness to receive COVID-19 vaccination. The PLWH who had a monthly income over 5,000 RMB (OR = 0.64, 95% CI: 0.45–0.92) and had a sex orientation as non-homosexual (OR = 0.67, 95% CI: 0.47–0.96) were associated with lower willingness to receive COVID-19 vaccination (Table 2).

**Table 2:** Univariate analysis and multivariable logistic regression on factors associated with COVID-19 vaccination willingness among PLWH in Wuhan, China (*N* = 556).

**Characteristics**	**Willing to receive COVID-19 vaccination**	**Unwilling to receive COVID-19 vaccination**	**Statistics**	***P***	**Univariable logistic analysis**		**Multivariable logistic regression**	
					**Crude OR (95%CI)**	***P***	**Adjusted OR (95%CI)**	***P***
Gender								
Male	312 (60.6)	203 (39.4)			ref.			
Female	26 (63.4)	15 (36.6)	0.13	0.721	1.13 (0.58–2.18)	0.721		
Age (Mean ± SD)					1.01 (0.99–1.02)	0.422		
Age group								
18–59	318 (60.8)	205 (39.2)			ref.			
≥60	20 (60.6)	13 (39.4)	0.001	0.982	0.99 (0.48–2.04)	0.982		
Marital status								
Married	100 (62.1)	61 (37.9)			ref.			
Single or Divorced or Widowed	238 (60.3)	157 (39.7)	0.17	0.684	0.93 (0.64–1.35)	0.684		
Educational level								
High school or lower	199 (63.2)	116 (36.8)			ref.			
Higher than high school	139 (57.7)	102 (42.3)	1.73	0.188	0.79 (0.56–1.12)	0.188		
Monthly income (RMB)								
≤ 5,000	191 (66.1)	98 (33.9)			ref.		ref.	
>5,000	147 (55.1)	120 (44.9)	7.23	0.008	0.63 (0.45–0.89)	0.008	0.64 (0.45–0.92)	0.015
Sexual orientation								
Homosexual	188 (64.8)	102 (35.2)			ref.		ref.	
Others	150 (56.8)	116 (43.2)	4.41	0.042	0.71 (0.50–0.99)	0.042	0.67 (0.47–0.96)	0.03
Comorbidities								
No	279 (58.0)	202 (42.0)			ref.		ref.	
Yes	59 (78.7)	16 (21.3)	11.62	0.001	2.67 (1.49–4.78)	0.001	2.07 (1.25–3.45)	0.005
ART								
No	10 (45.5)	12 (54.5)						
Yes	328 (61.4)	206 (38.6)	2.2	0.133	1.91 (0.81–4.50)	0.133		
The last CD4 result								
<350	79 (62.7)	47 (37.3)			ref.			
≥350	235 (60.3)	155 (39.3)			0.90 (0.60–1.37)	0.625		
Not test	24 (60.0)	16 (40.0)	0.250	0.883	0.76 (0.43–1.85)	0.759		
Sexual orientation disclosure								
No	220 (61.5)	138 (38.5)			ref.			
Yes	118 (59.5)	80 (40.5)	0.18	0.668	0.93 (0.65–1.32)	0.668		
Have taken other vaccines in the last 3 years								
No	298 (59.8)	200 (40.2)			ref.			
Yes	40 (69.0)	18 (31.0)	1.82	0.178	1.49 (0.83–2.68)	0.180		
HIV viral load								
Not detected	248 (60.5)	162 (39.5)			ref.			
Detected	90 (61.6)	56 (38.4)	0.06	0.806	1.05 (0.71–1.55)	0.806		
ART affected by COVID-19								
No	227 (56.6)	174 (43.4)			ref.		ref.	
Yes	111 (71.6)	44 (28.4)	10.56	0.001	2.134 (1.32-3.59)	0.001	2.04 (1.22–3.42)	0.007
Ever diagnosed with COVID-19								
No	333 (60.7)	215 (39.3)			ref.			
Yes	5 (62.5)	3 (37.5)	0.01	0.921	1.08 (0.26–4.55)	0.921		
Family members/friends diagnosed with COVID-19								
No	311 (60.1)	206 (39.9)			ref.			
Yes	27 (69.2)	12 (30.8)	1.25	0.263	1.49 (0.74–3.01)	0.266		
HIV positive members are more likely to get infected with SARS-CoV-2								
No	209 (59.9)	140 (40.1)			ref.			
Yes	129 (62.3)	78 (37.7)	0.32	0.570	1.11 (0.78–1.58)	0.57		
PLWH would be more serious if they were infected with SARS-CoV-2								
No	79 (51.6)	74 (48.4)			ref.		ref.	
Yes	259 (64.3)	144 (35.7)	7.34	0.007	1.73 (1.16–2.58)	0.007	1.67 (1.11–2.51)	0.014

### Reasons for Willingness or Unwillingness to Receive the COVID-19 Vaccination Among PLWH

There was a total of 364 PLWH willing to receive the COVID-19 vaccination. The top stated reasons were vaccination being a civic duty (68.6%), vaccines being effective (63.3%), and vaccines being safe (46.8%). This was followed by the convenience to take the vaccine (27.5%), recommended by people around (25.2%), free of charge (22.8%), and occupational requirement (9.8%) (Table 3).

**Table 3:** Reasons for willingness or unwillingness to receive COVID-19 vaccination among PLWH in Wuhan, China (*N* = 556).

	***N***	**%**
Willingness to receive COVID-19 vaccination: [can choose more than one]		
Vaccination is a civic duty	250	68.6
I believe that the vaccine is effective	230	63.3
I believe that the vaccine is safe	170	46.8
It is very convenient to take the vaccine	100	27.5
Recommended by people around	92	25.2
Free of charge	83	22.8
Occupational require	36	9.8
Others	13	3.6
Unwillingness to receive COVID-19 vaccination: [can choose more than one]		
I am concerned that the COVID-19 vaccine would affect my ART against HIV	144	75.2
I am concerned about the potential side effect	75	39.0
I'm concerned about the information leakage	65	33.6
I think COVID-19 vaccine may not be safe	55	28.9
I think COVID-19 vaccine may not be effective	47	24.3
There is no need to get vaccinated because COVID-19 has been controlled	15	7.8
I don't know the place where I could get vaccinated	5	2.8
Others	14	7.3

**Figure 2:**
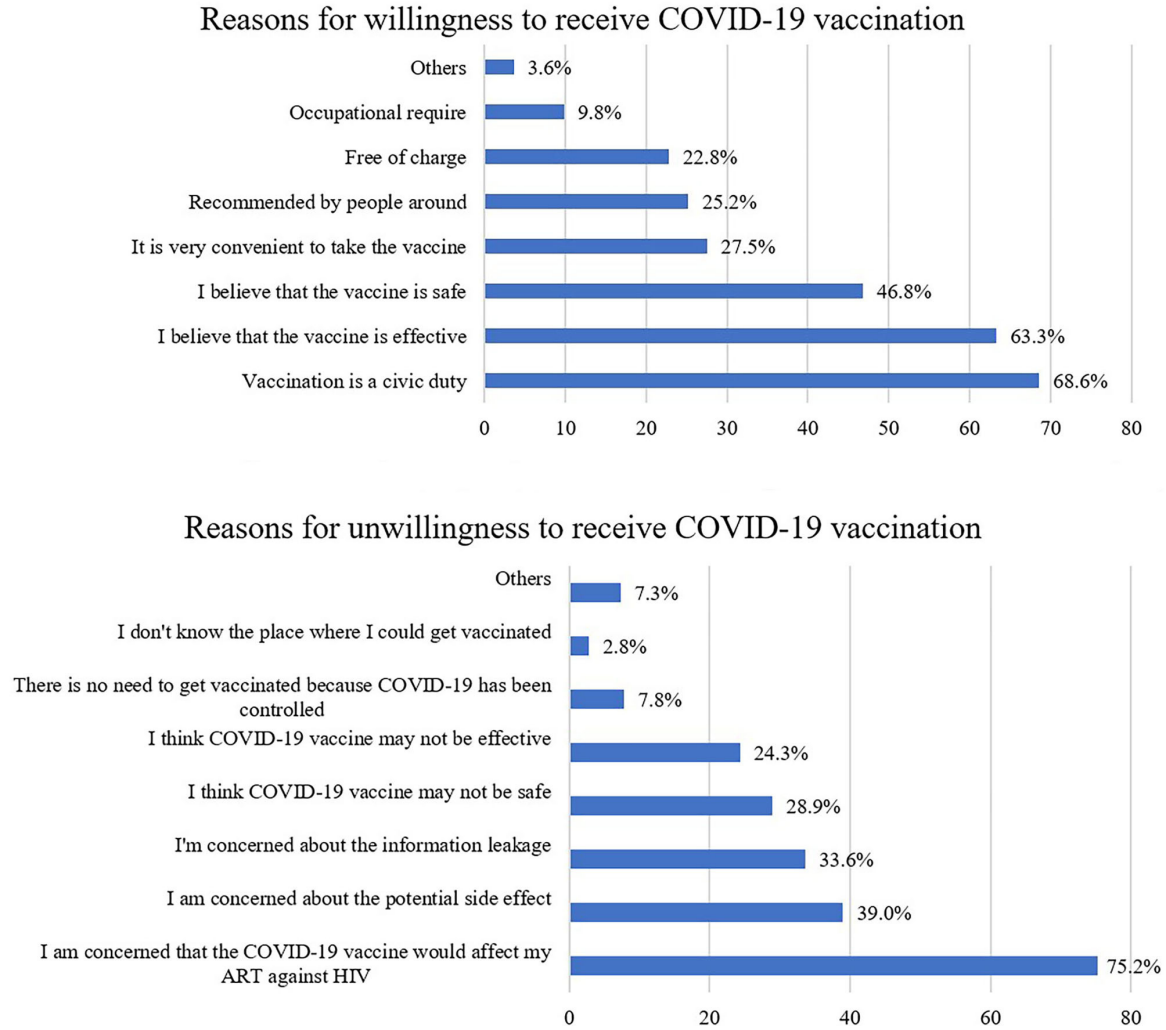
Proportion of reasons for willingness and unwillingness to receive COVID-19 vaccination among PLWH.

On the other hand, there were 192 PLWH unwilling to receive the COVID-19 vaccination. The top two reasons were that they were concerned that the vaccination would affect their ART (75.2%) and the concern about the potential side effects of the vaccine (39.0%). This was followed by the fear of information leakage (33.6%). Further, 28.9% of PLWH thought that COVID-19 vaccine might not be safe and 24.3% thought that COVID-19 vaccine might not be effective (Figure 2).

## Discussion

The central Chinese city of Wuhan, where the first outbreak of COVID-19, had been locked down for more than 2.5 months (19–22). The epidemic has significantly impacted people's emotional and social wellbeing for all populations and has brought more obstacles for the health care and the access to ART among PLWH. Vaccination would be one of the most important measures for controlling the epidemic, so it is essential to understanding the COVID-19 vaccination willingness among PLWH in Wuhan.

We found that PLWH had a low willingness to receive COVID-19 vaccination (65.5%), which is significantly lower than that in the general population in Wuhan, China (80.9%). This level was lower than that of PLWH in France (14) and the United States (13) as well as the general population in most parts of the world (23) and China (24, 25). But it was higher than that of PLWH from a nationwide survey in China (15) and also higher than that in south India (26). The comparison between the data from these two groups suggested that there was a more serious phenomenon of vaccine hesitancy among PLWH. Since there is a gap between willingness and actual uptake, COVID-19 vaccination coverage would be even lower without effective interventions (27). These findings indicated that it is urgent to implement more comprehensive and targeted interventions so as to promote COVID-19 vaccination among PLWH. We also found the majority of general population in Wuhan were willing to receive COVID-19 vaccination (80.9%), which was slightly lower than the willingness to receive COVID-19 vaccination among the public population in a nationwide survey in China (28), but higher than that observed in seven European countries (73.9%) (29). This result may reflect the strong demand for COVID-19 vaccination in the general population in Wuhan.

The results of multivariable logistic regression analyses showed that PLWH with comorbidities had higher COVID-19 vaccination willingness, similar results were found for other vaccines such as influenza (30). PLWH with comorbidities potentially had been already engaged in preventative practices. They had an appreciation for the benefits of preventative healthcare; therefore, they are more willing to vaccinate against a preventable illness. According to our result, vaccine education efforts should address vaccine complacency, particularly among PLWH without comorbidities.

Our study also suggested that PLWH who had idea that they would be more serious if they were infected with SARS-CoV-2 reported higher willingness. Studies have shown that perceived severity were positively correlated with the acceptability and uptake of vaccines, which is consistent with previous studies (31, 32). Past interventions that have included components targeting such perceptions have been successful in improving knowledge, attitudes/beliefs, and uptake of other vaccines (33, 34). The results indicated that interventions targeted at modifying such health beliefs about COVID-19 may lead to improved vaccination rates.

In addition, the results showed that PLWH with a monthly income of ≥5,000 RMB reported lower COVID-19 vaccination willingness, which is consistent with other studies (35) and they may think they will have more favorable living conditions to prevent COVID-19 infection. It suggested that communication and education strategies on vaccines should pay more attention for PLWH with higher income.

The main reasons for unwillingness to receive the COVID-19 vaccination among PLWH are concerns that ART would affect COVID-19 vaccination, the potential side effects, the safety and efficacy of the COVID-19 vaccines, which is consistent with various studies (36). According to the recommendation from the United Nations AIDS program (UNAIDS), the COVID-19 vaccine is safe for PLWH and could bring the same benefits as they bring to all individuals and communities, the COVID-19 vaccination should be recommended for PLWH regardless of their CD4 count and HIV viral load levels or even given priority to them (37). The national technical guideline for COVID-19 vaccination in China encourages PLWH to take up inactivated vaccines or recombinant subunit vaccines (38). Several studies have reported that the PLWH has a comparable immune response to COVID-19 vaccines and there are no serious adverse events after COVID-19 vaccination (11, 39). It is essential to design and develop health education programs to improve their perceptions and confidence in COVID-19 vaccines to improve COVID-19 vaccination coverage.

Some limitations should be mentioned. First, selection bias existed due to the potential limitation of enrollment methods and the generalizability of the results from this study should be done with caution. Second, this was a questionnaire-based cross-sectional survey, thus suffering from the inherent weaknesses of this type of study, such as recall bias. Third, this study focuses only on willingness to receive the COVID-19 vaccination among PLWH, however, a willingness to be vaccinated does not necessarily lead to the actual practice of vaccination. Further study is warranted to explore the vaccination situation of PLWH who are willing to receive the COVID-19 vaccine. In addition, the willingness to receive the COVID-19 vaccine would change as people's thoughts, decisions, and perceptions could change rapidly over time, a more comprehensive study at a different time will be conducted to compare with the results of this study next.

In conclusion, our study showed that PLWH in Wuhan reported a relatively low willingness to receive COVID-19 vaccination compared to the general population. PLWH with comorbidities, those who had idea that they would be more serious if they were infected with COVID-19 reported higher COVID-19 vaccination willingness, while PLWH with a monthly income of >5,000 RMB reported lower willingness. Targeted interventions such as enhancing health education should be conducted to enhance COVID-19 vaccine uptake among PLWH.

## Data Availability Statement

The raw data supporting the conclusions of this article will be made available by the authors, without undue reservation.

## Ethics Statement

The study was reviewed and approved by the Research and Ethics Committee of Zhongnan Hospital (2020062). Signed electronic informed consent forms were obtained from all participants involved in the study.

## Author Contributions

YL and KL conceived and designed this investigation and revised the manuscript. FM and SZh performed the data collection. SW analyzed the data. SW, ZX, and ZZ drafted the manuscript. FM, WG, SZo, and JL gave advise for the discussion part. All authors have read and approved the final version of the manuscript.

## Funding

This work was supported by the Philosophy and Social Science research Project in the Department of Education of Hubei Province (Grant No. 21G001), Medical Science and Technology Innovation Platform Support Project of Zhongnan Hospital, Wuhan University (PTXM2020008), Science and Technology Innovation Cultivation Fund of Zhongnan Hospital, Wuhan University (cxpy2017043), Medical Science Advancement Program (Basic Medical Sciences) of Wuhan University (TFJC2018004), and the Non-profit Central Research Institute Fund of Chinese Academy of Medical Sciences (2020-PT320-004).

## Conflict of Interest

The authors declare that the research was conducted in the absence of any commercial or financial relationships that could be construed as a potential conflict of interest.

## Publisher's Note

All claims expressed in this article are solely those of the authors and do not necessarily represent those of their affiliated organizations, or those of the publisher, the editors and the reviewers. Any product that may be evaluated in this article, or claim that may be made by its manufacturer, is not guaranteed or endorsed by the publisher.
