# Supplementary material for: COVID-19 Vaccination Willingness Among People Living With HIV in Wuhan, China
Source: Front Public Health. 2022 May 9;10:883453. doi: 10.3389/fpubh.2022.883453 (PMC9124942; doi:10.3389/fpubh.2022.883453)
Supplement: Supplementary file 1 [file Table_1.docx]

| Table S1. Univariate analysis and multivariable logistic regression on factors associated with COVID-19 vaccination willingness among all participants（N=1126） | | | | | | | | |
| --- | --- | --- | --- | --- | --- | --- | --- | --- |
| Characteristics | Willing to receive COVID-19 vaccination | Unwilling to receive COVID-19 vaccination | Statistics | *P* | Univariable logistic analysis | | Multivariable logistic regression | |
|  |  |  |  |  | Crude OR (95%CI) | *P* | Adjusted OR (95%CI) | *P* |
| Group |  |  |  |  |  |  |  |  |
| General population | 461(80.9) | 109(19.1) |  |  |  |  | ref. |  |
| PLWH | 338(60.8) | 218(39.2) | 55.103 | ＜0.001 | 0.36（0.28-0.48） | ＜0.001 | 0.29(0.21-0.41) | ＜0.001 |
| Gender |  |  |  |  |  |  |  |  |
| Male | 520(67.9) | 246(32.1) |  |  | ref. |  | ref. |  |
| Female | 279(77.5) | 81(22.5) | 10.987 | 0.001 | 1.63(1.22-2.18) | 0.001 | 1.25(0.90-1.73) | 0.178 |
| Age group |  |  |  |  |  |  |  |  |
| 18-59 | 756（71.0） | 309(29.0) |  |  | ref. |  | ref. |  |
| ≥60 | 43（70.5） | 18(29.5) | 0.007 | 0.934 | 0.98(0.0.55-1.72) | 0.934 | 0.90(0.44-1.83) | 0.763 |
| Marital status |  |  |  |  |  |  |  |  |
| Married | 366(74.7) | 124(25.3) |  |  | ref. |  | ref. |  |
| Single or Divorced or Widowed | 433(48.2) | 403(51.8) | 5.87 | 0.015 | 0.72(0.55-0.94) | 0.015 | 0.81(0.60-1.09) | 0.160 |
| Educational level |  |  |  |  |  |  |  |  |
| High school or lower | 355(76.2) | 114(23.8) |  |  | ref. |  | ref. |  |
| Higher than high school | 444(67.6) | 213(32.4) | 8.741 | 0.003 | 0.70(0.51-0.97) | 0.003 | 0.75(0.58-1.05) | 0.094 |
| Monthly income (RMB) |  |  |  |  |  |  |  |  |
| ≤5000 | 396（75.2） | 130(24.8) |  |  | ref. |  | ref. |  |
| ＞5000 | 403(67.2) | 197(32.8) | 8.965 | 0.003 | 0.67(0.52-0.87) | 0.003 | 0.79(0.60-1.06) | 0.110 |
| Comorbidities |  |  |  |  |  |  |  |  |
| No | 701(69.6) | 306(30.4) |  |  | ref. |  | ref. |  |
| Yes | 98(82.4) | 21(17.6) | 8.38 | 0.004 | 2.04(1.25-3.32) | 0.004 | 2.15(1.26-3.68) | 0.005 |
